# Understanding the pathways between prenatal and postnatal factors and overweight outcomes in early childhood: a pooled analysis of seven cohorts

**DOI:** 10.1038/s41366-023-01301-9

**Published:** 2023-04-03

**Authors:** Miaobing Zheng, Kylie D. Hesketh, Peter Vuillermin, Jodie Dodd, Li Ming Wen, Louise A. Baur, Rachael Taylor, Rebecca Byrne, Seema Mihrshahi, David Burgner, Mimi L. K. Tang, Karen J. Campbell

**Affiliations:** 1 Institute for Physical Activity and Nutrition, School of Exercise and Nutrition Sciences, Deakin University, Geelong, VIC Australia; 2 Barwon Health, Geelong, VIC Australia; 3 Discipline of Obstetrics and Gynaecology, The Robinson Research Institute, The University of Adelaide, Adelaide, SA Australia; 4 Faculty of Medicine and Health, The University of Sydney, Sydney, NSW Australia; 5 Department of Medicine, University of Otago, Dunedin, New Zealand; 6 School of Exercise and Nutrition Sciences, Faculty of Health, Queensland University of Technology, Victoria Park Road, Kelvin Grove, QLD Australia; 7 Department of Health Sciences, Faculty of Medicine, Health and Human Sciences, Macquarie University, Sydney, NSW Australia; 8 Murdoch Children’s Research Institute, Royal Children’s Hospital, Parkville, VIC Australia; 9 Department of Paediatrics, University of Melbourne, Parkville, VIC Australia

**Keywords:** Epidemiology, Risk factors

## Abstract

**Background/objectives:**

Childhood overweight and obesity are influenced by a range of prenatal and postnatal factors. Few studies have explored the integrative pathways linking these factors and childhood overweight. This study aimed to elucidate the integrative pathways through which maternal pre-pregnancy body mass index (BMI), infant birth weight, breastfeeding duration, and rapid weight gain (RWG) during infancy are associated with overweight outcomes in early childhood from ages 3 to 5 years.

**Subjects/methods:**

Pooled data from seven Australian and New Zealand cohorts were used (*n* = 3572). Generalized structural equation modelling was used to examine direct and indirect associations of maternal pre-pregnancy BMI, infant birth weight, breastfeeding duration, and RWG during infancy with child overweight outcomes (BMI *z*-score and overweight status).

**Results:**

Maternal pre-pregnancy BMI was directly associated with infant birth weight (β 0.01, 95%CI 0.01, 0.02), breastfeeding duration ≥6 months (OR 0.92, 95%CI 0.90, 0.93), child BMI *z*-score (β 0.03, 95%CI 0.03, 0.04) and overweight status (OR 1.07, 95%CI 1.06, 1.09) at ages 3–5 years. The association between maternal pre-pregnancy BMI and child overweight outcomes was partially mediated by infant birth weight, but not RWG. RWG in infancy exhibited the strongest direct association with child overweight outcomes (BMI *z*-score: β 0.72, 95%CI 0.65, 0.79; overweight status: OR 4.49, 95%CI 3.61, 5.59). Infant birth weight was implicated in the indirect pathways of maternal pre-pregnancy BMI with RWG in infancy, breastfeeding duration, and child overweight outcomes. The associations between breastfeeding duration (≥6 months) and lower child overweight outcomes were fully mediated by RWG in infancy.

**Conclusions:**

Maternal pre-pregnancy BMI, infant birth weight, breastfeeding duration and RWG in infancy act in concert to influence early childhood overweight. Future overweight prevention interventions should target RWG in infancy, which showed the strongest association with childhood overweight; and maternal pre-pregnancy BMI, which was implicated in several pathways leading to childhood overweight.

## Introduction

Overweight and obesity are global health concerns affecting populations of all ages. These conditions have become increasingly prevalent in young children, with over 39 million children under the age of five being classified as overweight or obese in 2020 [1]. Overweight and obesity risk develops early in life, often persists across the lifespan, and is associated with a range of adverse health consequences that have far-reaching and lifelong impact [1, 2]. Early prevention of overweight and obesity is therefore a public health priority. Understanding the early origins of overweight and obesity is imperative for planning preventive strategies and interventions to reduce childhood overweight and obesity prevalence.

Several reviews have critically summarized the early risk factors associated with childhood overweight and obesity [3–5]. A robust body of evidence suggests that prenatal and postnatal factors such as high maternal pre-pregnancy body mass index (BMI) [6], high infant birth weight [7], rapid weight gain (RWG) during infancy [8] and short breastfeeding duration or no breastfeeding [9] are notable contributing factors to childhood overweight and obesity. However, the collective effect of these factors on childhood overweight and obesity remains unknown. Most observational studies used multiple regression analyses to identify determinants of childhood overweight and obesity, where several factors of interest were included in the same model as predictors of overweight and obesity [3–5]. These models do not capture the interplay between individual determinants of overweight and obesity and are prone to multicollinearity when predictor variables are correlated. For instance, longer breastfeeding duration is associated with lower risk of RWG in infancy [10]. Furthermore, some determinants may lie on the causal pathways between other determinants and overweight/obesity. However, the underlying mechanisms through which each determinant influences overweight and obesity cannot be examined by linear regression models. For example, higher maternal pre-pregnancy BMI is associated with higher childhood overweight and obesity risk potentially via the mediating effect of infant birth weight [6]. Moreover, there might be multiple pathways underlying the association between infant birth weight and subsequent risk of overweight and obesity [11]. For example, high infant birth weight is associated with increased overweight and obesity risk [11, 12], but catch-up growth, that is commonly observed in low birth weight infants, may predispose to subsequent overweight and obesity risk [13]. Furthermore, some determinants of overweight and obesity may occur sequentially over time (i.e., infant birth weight, rapid weight gain during infancy, breastfeeding duration), and simply putting them into the same model does not account for the longitudinal nature. Considering the complex interrelationships among the early determinants of overweight and obesity, it is critical to disentangle the respective sequential and integrative pathways through which these prenatal and postnatal factors lead to childhood overweight and obesity. Such information will help inform public health policy and practice by clarifying when and what to target in early overweight and obesity prevention efforts and strategies.

Structural equation modeling (SEM) enables simultaneous estimation of relationships among a complex network of variables and computation of both direct and indirect effects (i.e., mediational effect), enabling insights into the etiology of overweight and obesity. A small number of studies have used SEM to estimate the casual underpinning of childhood overweight and obesity, but these studies were limited to cross-sectional design, small sample size and inclusion of older children aged 5 years and over [14, 15]. No studies to date appear to have explored the integrative pathways between prenatal, postnatal factors (i.e., maternal BMI, infant birth weight RWG in infancy and breastfeeding duration) and early childhood overweight and obesity. Therefore, this study explored the pathways linking prenatal and postnatal factors and overweight outcomes in early childhood, and elucidated the underlying mechanisms via examination of the direct and indirect relationships using SEM.

## Methods

Data were from seven Australian and New Zealand cohorts: BIS (Barwon Infant Study) [16], HB (Healthy Beginnings) [17–19], InFANT (Infant Feeding Activity and Nutrition Trial) [20, 21], InFANT Extend [22], LIMIT [23, 24], Nourish [25, 26], and POI (Prevention of Obesity in Infancy) [27, 28]. All cohorts collected information on a range of child and maternal factors and had repeated anthropometric measurements from birth up to age 5 years. BIS was a population-based birth cohort. The remaining six studies were randomized controlled trials aiming to prevent childhood obesity, with subsequent follow-up into early childhood without intervention to test sustainability of the intervention effects up to age 5 years. Five of the six studies (HB, InFANT, InFANT Extend, Nourish, POI) involved parent education sessions prenatally or in infancy to promote healthy lifestyle behaviors including healthy eating and active play. The LIMIT trial was a pregnancy intervention that involved lifestyle advice on diet and exercise for pregnant women. Apart from Nourish and POI which included full term infants only, the remaining five cohorts have no restrictions on gestational age at the baseline recruitment. These six studies did not demonstrate any significant intervention effect on BMI *z*-score at ages 3–5 years. Details of each study have been reported previously [29]. All studies obtained ethical approval from relevant ethics committees and informed consent from participants. The ethics approval for the current pooled analysis has been granted by Deakin University Human Research Ethics Committee (project number: 2023-033).

### Anthropometric measurements

In all cohorts, length and weight at birth were extracted from child health records. Trained staff used standardized protocols to measure child length and weight at two time points: one in the first year of life and the other between 3 and 5 years of age. Age- and sex-specific weight- and body mass index (BMI)-for-age *z*-scores were derived using WHO growth standards [30]. RWG in infancy was defined as the difference in weight-for-age *z*-score between birth and infancy >0.67, which is clinically equivalent to crossing one centile line in a growth chart [8, 31]. Overweight status in early childhood was defined using age- and sex-specific International Obesity Task Force cut-offs [32], which is the recommended definition for children over 2 years in cohort studies [33].

### Maternal and postnatal factors

Maternal age, pre-pregnancy height and weight, were reported by parents via questionnaire at baseline of each study. Maternal age was reported in years. Maternal pre- or early-pregnancy BMI was calculated as weight in kilograms by height in meters squared (kg/m^2^). All cohorts assessed breastfeeding using questionnaires at multiple time points during infancy. Duration of any breastfeeding in months was derived and categorized into <6 vs ≥6 months. Six of the seven cohorts collected maternal education (tertiary education vs non-tertiary education). All variables were coded in a uniform way to enable pooled analysis.

### Statistical analysis

All statistical analyses were conducted in Stata 16 (StataCorpLLC) with statistical significance set at *P* < 0.05. Characteristics of each cohort were assessed using descriptive analysis. Generalized structural equation models (GSEM) [34] were used to evaluate the integrative pathways linking prenatal and postnatal factors and overweight outcomes (BMI *z*-score and overweight status) in early childhood. GSEM estimates multiple pathways simultaneously, allowing estimation of direct, indirect, and total effects. Maximum likelihood method was used to estimate model parameters. Compared to SEM, GSEM enables inclusion of both continuous and categorical variables. Separate GSEMs were fitted for BMI *z*-score and overweight status in early childhood. Continuous variables were analyzed with Gaussian family and identity link function. Binary variables: RWG in infancy (yes vs no), breastfeeding duration (≥6 vs <6 months), and overweight status (yes vs no) were analyzed using binomial family with logit link.

Hypothesized pathways were established based on existing well-documented associations (Supplementary fig. 1). Model fit was assessed based on likelihood ratio test, Akaike and Bayesian information criteria and statistical significance of path coefficient and clinical interpretability [35]. A series of models were tested and the final model with the following pathways exhibited the best model fit: maternal pre-pregnancy BMI as a predictor for infant birth weight, RWG in infancy, breastfeeding duration, and early childhood overweight outcomes; infant birth weight as a predictor for RWG in infancy, breastfeeding duration and early childhood overweight outcomes; RWG in infancy as a predictor for early childhood overweight outcomes; breastfeeding as a predictor for early childhood overweight outcomes.

Moreover, we assessed the direct, indirect, and total effects for four main pathways (exposure → outcomes):

- maternal pre-pregnancy BMI → early childhood overweight outcomes and respective indirect effects via infant birth weight, RWG in infancy, and breastfeeding duration
- maternal pre-pregnancy BMI → RWG in infancy and indirect effects via infant birth weight, breastfeeding duration
- infant birth weight → early childhood overweight outcomes and indirect effects via RWG in infancy, breastfeeding duration
- breastfeeding duration → early childhood overweight outcomes and indirect effect via RWG in infancy
- maternal pre-pregnancy BMI → breastfeeding duration and indirect effect via infant birth weight

The indirect effect was calculated using the product method and total effect was calculated as the sum of direct and indirect effect [36]. Indirect and total effects were calculated using the “nlcom” command [37]. Bootstrapping with 1000 samples was conducted using “program” command to obtain bias-corrected and normal-based confidence intervals for all estimated effects. The direct, indirect, and total effects were considered significant if the bootstrapped confidence interval did not include zero. The interpretation of the mediational pathways was informed by MacKinnon, et al. [38].

Child sex and maternal age were tested as potential moderators, but no evidence of moderation was found. Therefore, child sex and maternal age were included as covariates in the GSEM. To account for cohort specific effects and variation in age when RWG in infancy and early childhood overweight outcomes were assessed, we also controlled for cohort, ages at infancy and early childhood assessments. As six of seven cohorts involved intervention during the early stage of the study, intervention allocation was also included as a covariate.

Additional analyses were conducted to add maternal education in the model as a moderator among six of the seven cohorts that had data on maternal education. No evidence of moderation was found, thus maternal education was included as a covariate along with aforementioned covariates.

### Sensitivity analyses

In order to test whether the variations in the timing of assessment in infancy influence the results, sensitivity analyses were conducted excluding cohorts that had infancy assessment before 1 year of age (InFANT, InFANT Extend, LIMT). Similarly, analyses separating cohorts with outcome assessment at ages 3–4 years (BIS, InFANT Extend, LIMIT) and age 5 years (HB, InFANT, Nourish, POI) were performed to examine whether variations in timing of assessment in early childhood influenced the results. Additional sensitivity analyses leaving one cohort out were conducted to assess the potential effect of cohort heterogeneity in the pooled results.

## Results

### Sample characteristics

A total of 3572 participants with complete data on maternal pre-pregnancy BMI, infant birth weight, breastfeeding duration, RWG in infancy and child overweight outcomes in early childhood were included in the current analyses. Characteristics of the seven cohorts are presented in Table 1. All cohorts had comparable cohort characteristics apart from LIMIT that exclusively comprised women with overweight and obesity. Data on gestational age were collected in five of the seven cohorts, most children were full term ranging from 88.6%(InFANT) to 100% (POI). Across cohorts, the proportion of RWG in infancy ranged from 21.2 to 36.0%, and the proportion of overweight/obesity in early childhood spanned from 11.4 to 22.4%.

**Table 1:** Characteristics of the seven cohorts^a^.

	BIS	HB	InFANT	InFANT Extend (*n* = 243)	LIMIT (*n* = 1230)	Nourish	POI
	(*n* = 562)	(*n* = 275)	(*n* = 352)			(*n* = 395)	(*n* = 515)
Intervention allocation							
Control	n/a	49.5%	49.3%	49.2%	49.8%	52.0%	51.2%
Intervention		50.5%	50.7%	50.8%	50.2%	48.0%	48.8%
*Maternal factors*							
Maternal age (years)	31.8 ± 4.3	27.4 ± 5.3	32.5 ± 4.1	32.5 ± 4.2	30.0 ± 5.3	31.9 ± 5.0	32.7 ± 4.6
Maternal education							
Non-tertiary	44.3%	32.5%	40.3%	38.7%	n/a	30.1%	31.8%
Tertiary	55.7%	67.5%	59.7%	61.3%		69.9%	68.2%
Maternal pre-pregnancy BMI (kg/m^2^)	25.2 ± 5.1	25.3 ± 5.4	24.1 ± 5.2	24.2 ± 4.6	32.2 ± 5.8	25.7 ± 5.2	25.1 ± 4.9
Normal weight	57.8%	56.4%	67.1%	63.8%	0.0%	53.7%	60.2%
Overweight	27.1%	28.0%	21.3%	26.3%	44.2%	29.4%	26.2%
Obesity	15.1%	15.6%	11.6%	9.9%	55.9%	17.0%	13.6%
*Child factors*							
Child age at assessment in infancy (years)	1.1 ± 0.1	1.0 ± 0.0	0.8 ± 0.1	0.8 ± 0.2	0.6 ± 0.2	1.1 ± 0.1	1.0 ± 0.0
Child age at assessment in early childhood (years)	4.1 ± 0.2	5.0 ± 0.1	5.1 ± 0.2	3.2 ± 0.1	3.3 ± 0.5	5.0 ± 0.0	5.0 ± 0.0
Gestational age							
Premature (<37 weeks)	4.1%	n/a	11.4%	7.9%	5.4%	n/a	0
Full term (≥37 weeks)	95.9%		88.6%	92.1%	94.6%		100%
Child sex							
Boys	53.7%	53.1%	52.3%	50.2%	49.6%	48.1%	51.8%
Girls	46.3%	46.9%	47.7%	49.8%	50.4%	51.9%	48.2%
Infant birth weight (kg)	3.5 ± 0.5	3.4 ± 0.6	3.3 ± 0.6	3.3 ± 0.6	3.5 ± 0.5	3.5 ± 0.4	3.6 ± 0.5
<2.5 kg	2.5%	5.5%	6.8%	7.0%	3.3%	0.0%	0.8%
2.5–4 kg	79.7%	84.0%	82.4%	83.5%	79.8%	89.6%	83.1%
≥4 kg	17.8%	10.6%	10.8%	9.5%	16.9%	10.4%	16.1%
Breastfeeding duration							
<6 months	34.2%	56.0%	33.3%	24.3%	44.4%	29.1%	25.1%
≥6 months	65.8%	44.0%	66.8%	75.7%	55.6%	70.9%	75.0%
Infant rapid weight gain							
Yes	25.6%	36.0%	29.0%	30.9%	23.2%	29.1%	21.2%
No	74.4%	64.0%	71.0%	69.1%	76.8%	70.9%	78.8%
Child BMI *z*-score in early childhood	0.2 ± 1.3	0.6 ± 1.0	0.5 ± 1.0	0.8 ± 0.9	0.8 ± 1.0	0.4 ± 0.9	0.5 ± 0.9
Underweight/normal weight	88.6%	78.6%	84.1%	85.6%	77.6%	87.9%	84.7%
Overweight/obesity	11.4%	21.5%	15.9%	14.4%	22.4%	12.2%	15.3%

Values are presented as % or mean ± standard deviation, n/a: not available.

^a^*BIS* Barwon Infant Study, *HB* Healthy beginnings, *INFANT* Infant Feeding Activity and Nutrition Trial, *POI* Prevention of Obesity in Infancy trial.

### Direct pathways

**Fig. 1:**
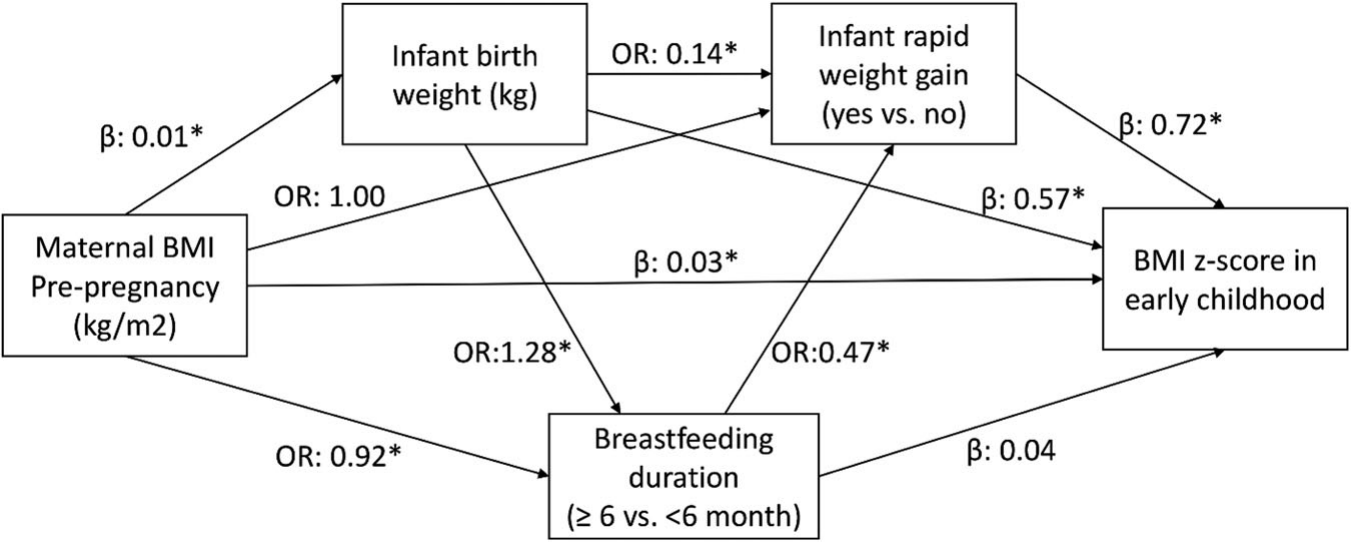
Pathways linking prenatal and postnatal factors and body mass index (BMI) *z*-score in early childhood. Adjusted for cohort, ages at infancy and early childhood assessments, maternal age, child sex, and intervention allocation. Effect sizes are direct effect. β-coefficients are presented for continuous outcomes and odds ratios are presented for categorical outcomes. Asterisk (*) indicates *P* < 0.001.

**Fig. 2:**
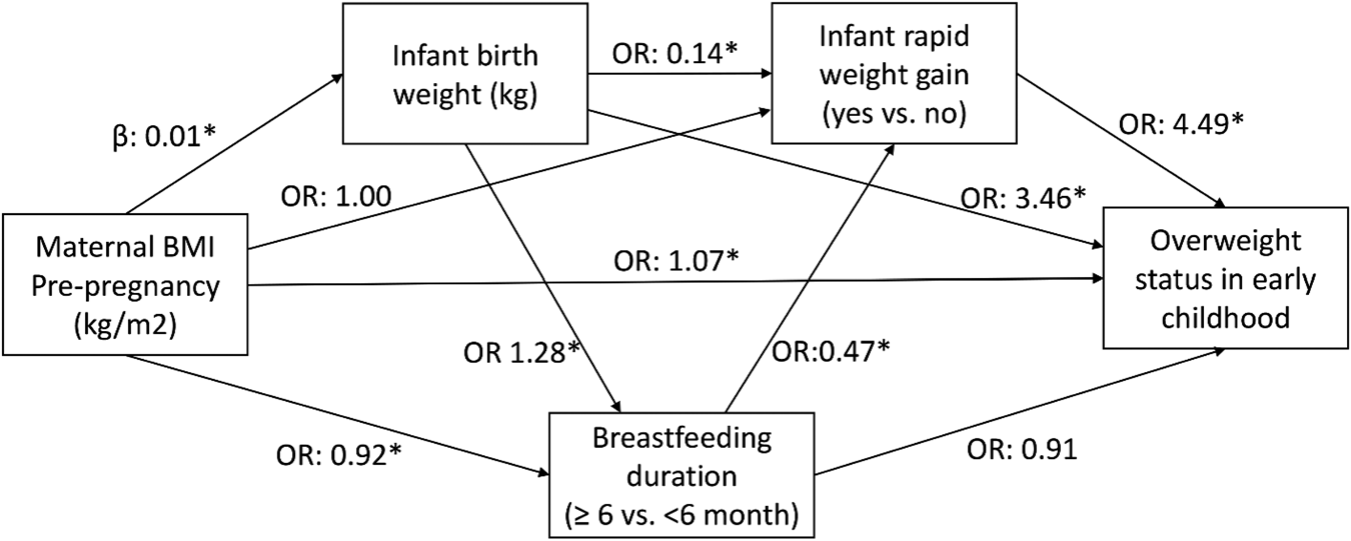
Pathways linking prenatal and postnatal factors and overweight status in early childhood. Effect sizes are direct effect. Adjusted for cohort, ages at infancy and early childhood assessments, maternal age, child sex, and intervention allocation. β-coefficients are presented for continuous outcomes and odds ratios are presented for categorical outcomes. Effect sizes are direct effect. Asterisk (*) indicates *P* < 0.001.

Maternal pre-pregnancy BMI was directly associated with infant birth weight (β^DE^ 0.01, 95%CI 0.01, 0.01), breastfeeding duration ≥6mo (OR^DE^ 0.92, 95%CI 0.90, 0.93), child BMI *z*-score (β^DE^ 0.03, 95%CI 0.03, 0.04) and overweight status (OR^DE^ 1.07, 95%CI 1.06, 1.09). One unit (kg/m^2^) increase in maternal pre-pregnancy BMI was associated with 0.01 kg increase in infant birth weight, 8% lower odds of having breastfeeding duration ≥6 months, 0.03 unit increase in BMI *z*-score and 7% increased odds of being overweight. Infant birth weight was found to have a direct association with RWG in infancy (OR^DE^ 0.14 95%CI 0.11, 0.17), breastfeeding duration ≥6mo (OR^DE^1.28, 95%CI 1.11, 1.47), child BMI *z*-score (OR^DE^ 0.57, 95%CI 0.51, 0.64), and overweight status (OR^DE^ 3.46, 95%CI 2.76, 4.34). A direct association was found between RWG in infancy and both higher BMI *z*-score (β^DE^ 0.72, 95%CI 0.65, 0.79) and higher odds of overweight (OR^DE^ 4.49, 95%CI 3.61, 5.59) in early childhood. Longer breastfeeding duration ≥6mo directly lowered the risk of RWG in infancy by 53% (OR^DE^ 0.47, 95%CI 0.40, 0.56) (Figs. 1 and 2).

### Indirect or mediational pathways

The association between higher maternal pre-pregnancy BMI and both higher child BMI *z*-score and odds of overweight were partially mediated by infant birth weight ((β^IE^ 0.01, 95%CI 0.01, 0.01; OR^IE^ 1.01, 95%CI 1.00, 1.02 respectively), but not RWG in infancy or breastfeeding duration (Tables 2 and 3).

**Table 2:** Total, direct and indirect effect among the pathways between prenatal (maternal pre-pregnancy BMI) and postnatal factors (infant birth weight, infant rapid weight gain, breastfeeding duration) and BMI *z*-score in early childhood: pooled effect from seven Australian and New Zealand cohorts.

	β or Odds ratio	95%CI		*P* value
Maternal pre-pregnancy BMI → Child BMI *z*-core				
Total effect	0.04	0.03	0.05	<0.001
Direct effect	0.03	0.03	0.04	<0.001
Indirect effect				
via infant birth weight	0.01	0.01	0.01	<0.001
via infant rapid weight gain	0.002	−0.01	0.01	0.70
via breastfeeding duration	−0.003	−0.01	0.003	0.28
Maternal pre-pregnancy BMI → Rapid weight gain in infancy (yes vs no)				
Total effect	1.05	1.02	1.07	<0.001
Direct effect	1.00	0.99	1.02	0.70
Indirect effect				
via infant birth weight	0.98	0.97	0.98	<0.001
via breastfeeding duration	1.07	1.05	1.09	<0.001
Maternal pre-pregnancy BMI → Breastfeeding duration (≥6 vs < 6 months)				
Total effect	0.92	0.91	0.93	<0.001
Direct effect	0.92	0.90	0.93	<0.001
Indirect effect				
via infant birth weight	1.00	1.00	1.01	0.002
Infant birth weight→ Child BMI *z*-score				
Total effect	−0.84	−1.03	−0.65	<0.001
Direct effect	0.57	0.51	0.64	<0.001
Indirect effect				
via infant rapid weight gain	−1.42	−1.62	−1.22	<0.001
via breastfeeding duration	0.01	−0.01	0.03	0.33
Breastfeeding duration(≥6 vs <6 months)→ Child BMI *z*-score				
Total effect	−0.50	−0.65	−0.34	<0.001
Direct effect	0.04	−0.03	0.11	0.28
Indirect effect				
via infant rapid weight gain	−0.54	−0.65	−0.34	<0.001

Adjusted for cohort, ages at infancy and early childhood assessments, maternal age, child sex, and intervention allocation. β-coefficients are presented for continuous outcome (BMI *z*-score), and odds ratios are presented for categorical outcomes.

*CI* confidence interval.

**Table 3:** Total, direct and indirect effect among the pathways between prenatal (maternal pre-pregnancy BMI) and postnatal factors (infant birth weight, infant rapid weight gain, breastfeeding duration) and overweight status in early childhood: pooled effect from seven Australian and New Zealand cohorts.

	Odds ratio	95%CI		*P* value
Maternal pre-pregnancy BMI → Child overweight status (yes vs no)				
Total effect	1.10	1.07	1.14	<0.001
Direct effect	1.07	1.06	1.09	<0.001
Indirect effect				
via infant birth weight	1.01	1.01	1.02	<0.001
via infant rapid weight gain	1.00	0.98	1.03	0.70
via breastfeeding duration	1.01	0.99	1.03	0.38
Maternal pre-pregnancy BMI → Rapid weight gain in infancy (yes vs no)				
Total effect	1.05	1.02	1.07	<0.001
Direct effect	1.00	0.99	1.02	0.70
Indirect effect				
via infant birth weight	0.98	0.97	0.98	<0.001
via breastfeeding duration	1.07	1.05	1.09	<0.001
Maternal pre-pregnancy BMI → Breastfeeding duration (≥6 vs < 6 months)				
Total effect	0.92	0.91	0.93	<0.001
Direct effect	0.91	0.90	0.93	<0.001
Indirect effect				
via infant birth weight	1.00	1.00	1.01	0.002
Infant birth weight→ Child overweight status (yes vs no)				
Total effect	0.18	0.11	0.28	<0.001
Direct effect	3.46	2.76	4.34	<0.001
Indirect effect				
via infant rapid weight gain	0.05	0.03	0.09	<0.001
via breastfeeding duration	0.98	0.93	1.03	0.41
Breastfeeding duration(≥6 vs <6 months)→ Child overweight status (yes vs no)				
Total effect	0.30	0.21	0.43	<0.001
Direct effect	0.91	0.74	1.12	0.37
Indirect effect				
via infant rapid weight gain	0.33	0.24	0.45	<0.001

Adjusted for cohort, ages at infancy and early childhood assessments, maternal age, child sex, and intervention allocation. Odds ratios are presented for categorical outcomes.

*CI* confidence interval.

Higher maternal pre-pregnancy BMI was associated with higher risk of RWG in infancy (OR^TE^ 1.05, 95%CI 1.02, 1.07) via indirect effects of breastfeeding duration and infant birth weight. Breastfeeding duration explained most of the association (OR ^IE^ 1.07, 95%CI 1.05, 1.09). Conversely, infant birth weight (OR^IE^ 0.98, 95%CI 0.97, 0.98) demonstrated a weak suppression effect (inconsistent mediation) as indicated by opposite direct (OR^DE^ > 1) and indirect effects (OR ^IE^ < 1).

The association between higher maternal BMI and lower odds of breastfeeding duration ≥6 months (OR^TE^ 0.92, 95%CI 0.91, 0.93) was slightly suppressed by infant birth weight with OR^IE^ > 1.

For the association between higher infant birth weight and higher child BMI *z*-score or odds of overweight, RWG in infancy was a mediator whereas breastfeeding duration was not. RWG in infancy showed a suppression effect as the indirect effect (β^IE^ −1.42, 95%CI −1.62, −1.22; OR^IE^ 0.05, 95%CI 0.03, 0.09) and direct effect (^DE^ 0.57, 95%CI 0.51, 0.64; OR^DE^ 3.46, 95%CI 2.76, 4.34) were in opposite directions.

The associations between longer breastfeeding duration ≥ 6mo and lower child BMI *z*-score (β^TE^ −0.50 95%CI −0.65, −0.34) or odds of overweight (OR^TE^ 0.30, 95%CI 0.21, 0.43) were fully mediated by RWG in infancy (β^IE^ −0.54, 95%CI−0.65, −0.34; OR^IE^ 0.33, 95%CI 0.24, 0.45) as evidenced by the non-significant direct effect.

Additional analysis among six cohorts with inclusion of maternal education as a covariate included 2342 participants. A direct association between maternal BMI and RWG in infancy, was revealed, but the path coefficient was small (OR^DE^ 1.02, 95%CI 1.00, 1.04). Moreover, RWG in infancy partially mediated the association between maternal pre-pregnancy BMI and child BMI *z*-score or overweight status. All other paths were in the same direction with path coefficients of similar magnitude (Supplementary Tables 1 and 2).

### Sensitivity analyses

Sensitivity analyses leaving some cohorts out to assess variations in timing of assessment during infancy and early childhood and cohort heterogeneity showed similar results to the pooled results from seven cohorts. All associations were in the same direction with similar magnitude of effects. Analyses were presented in Supplementary File.

## Discussion

Capitalizing upon data from seven Australian and New Zealand cohorts, this study investigated the integrative pathways linking maternal pre-pregnancy BMI, infant birth weight, RWG in infancy, breastfeeding duration, and child overweight outcomes in early childhood. Maternal pre-pregnancy BMI, infant birth weight and RWG in infancy showed direct positive associations with child overweight outcomes, with RWG in infancy demonstrating the strongest association. The associations between maternal pre-pregnancy BMI and child overweight outcomes was partially mediated by infant birth weight. Maternal pre-pregnancy BMI also showed a direct inverse association with breastfeeding duration (≥6 month), and an indirect association with RWG in infancy. The associations between breastfeeding duration and child overweight outcomes were fully mediated by RWG in infancy.

To our knowledge, this is the first study to use path analysis to elucidate the contributions of prenatal and postnatal factors on child overweight outcomes simultaneously in early childhood. The few previous studies that have used path analysis to investigate determinants of childhood obesity, have mostly used cross-sectional data from children aged 5 years and over [39, 40] and are of small sample size [14, 15, 41–43]. Moreover, those studies focused on early lifestyle behaviors (i.e., dietary intake, physical activity, and screen time) and/or family and environmental factors (i.e., family structure and food environment) without accounting for prenatal, birth and early life factors [14, 15, 39–43]. Only one US study involved data in early childhood and assessed the cross-sectional pathways linking gestational weight gain, infant birth weight, breastfeeding duration and overweight outcomes among children aged 0–6 years [44]. Consistent with our study findings, that study reported infant birth weight showed positive direct effects on both breastfeeding duration and child BMI *z*-score [44].

Higher maternal pre-pregnancy BMI has been identified as a risk factor for childhood obesity, possibly via alterations in metabolism in utero and shared genetic propensity, resulting in elevated susceptibility to obesity later in life [45, 46]. Our study supports these previous findings, and further clarified that infant birth weight, a marker of the in utero environment, partially explained the association between higher maternal pre-pregnancy BMI and higher childhood obesity risk. Moreover, RWG in infancy and breastfeeding duration did not mediate the association. It’s worth noting that the effect sizes of the associations between maternal pre-pregnancy BMI and childhood overweight outcomes were small.

Our study extends the current understanding of the association between maternal pre-pregnancy BMI and breastfeeding duration. Evidence has shown women with overweight or obesity are more likely to experience delayed lactogenesis (milk production), poor milk supply, breastfeeding difficulties, low breastfeeding intention and postpartum depression which in turn contribute to early cessation of breastfeeding [47]. Extending these findings, our study revealed that higher infant birth weight suppressed (inconsistent mediation) the inverse association between higher maternal pre-pregnancy BMI and shorter breastfeeding duration. Results from a US national survey found that infants with birth weight ≥2.5 kg had higher breastfeeding duration than those with low birth weight <2.5 kg [48]. It is conceivable that low birth weight infants may encounter more breastfeeding difficulties that impedes prolonged breastfeeding than normal birth weight infants [49].

Few studies have documented the associations between maternal pre-pregnancy BMI and RWG in infancy. A novel finding observed in our study was that the association between higher maternal pre-pregnancy BMI and higher risk of RWG in infancy was attributable to indirect pathways via breastfeeding duration (a mediator) and infant birth weight (a suppressor) [38]. Higher maternal BMI predicted higher risk of RWG through lowering the odds of breastfeeding duration ≥6 month. In contrast, higher infant birth weight suppressed the association between maternal pre-pregnancy BMI and risk of RWG in infancy. In accordance with our findings, a small US cohort found no direct association between maternal pre-pregnancy BMI and RWG in infancy [50]. Given the limited studies, further investigations are needed to understand how maternal pre-pregnancy BMI influences RWG in infancy, given this is a key predictor of later weight status.

In line with findings from previous meta-analyses, our study revealed that higher infant birth weight was directly associated with higher childhood BMI *z*-score [11, 12]. We also found this direct association was suppressed by RWG in infancy whereby higher infant birth weight was associated with lower risk of RWG in infancy, and subsequentially predicted lower childhood overweight outcomes. Our finding supports the view that high birth weight and RWG in infancy act on different pathways to increase obesity risk. Indeed, a 2018 systematic review found the association between RWG in infancy and later obesity risk remained after adjusting for birth weight in most studies [8]. This highlights the vital independent role of both infant birth weight and RWG in infancy in obesity development. Emerging evidence suggests that RWG (i.e., catch-up growth) in low birth weight infants may increase the risk of obesity, calling into questions pertaining to the benefits of RWG in low-birth-weight infants [13]. Studies with greater number of low birth weight infants are required to evaluate optimal growth in low birth weight infants.

The protective effects of longer breastfeeding duration on reducing obesity risk in children have been widely recognized. A growing number of studies have found that breastfed infants exhibited slower growth patterns during infancy than non-breastfed infants [51, 52], which may protect against the development of childhood obesity. Our study corroborates this, showing that the beneficial effect of breastfeeding duration ≥6 month in lowering subsequent BMI *z*-score and overweight risk was fully mediated by RWG in infancy. It is postulated that nutritional composition of breast milk may play a role in protecting against obesity. For example, breast milk contains hormones involved in appetite and energy balance regulation such as leptin, ghrelin [53].

### Strengths/limitations

The current study has several strengths and limitations. Pooled data from seven cohorts maximizes the sample size to facilitate use of complex GSEM to evaluate the interrelationship among multiple factors simultaneously, and elucidates mechanistic pathways through examination of total, direct and indirect pathways. The graphical illustration of the direct pathways in GSEM reveals insightful information on the relative contribution of each factor in childhood overweight outcomes. The use of pooled data also enhances the overall generalizability of the current study finding to the wider Australian and New Zealand populations. Six of the seven studies were longitudinal cohorts that started as early childhood or pregnancy obesity interventions and included a higher proportion of women with tertiary education, which may limit study generalizability. However, sensitivity analysis shows controlling for maternal education did not influence the study findings. Because of the pooled analysis, only variables that are available in all seven or six cohorts were included the present analyses, precluding the investigation of other factors that might be implicated in the etiology of childhood obesity such as maternal gestational weight gain, maternal ethnicity, maternal smoking, parity, birth order, breastfeeding exclusivity, and other socioeconomic variables. Unmeasured and residual confounding is possible. Nonetheless, inclusion of RWG in infancy, a pivotal risk factor of childhood obesity, in the GSEM is particularly valuable for evaluating pathways underlying early childhood obesity. The observational nature of the study limits causal inferences, although GSEM offers robust longitudinal evidence verifying the plausibility of the hypothesized causal pathways. Also, prenatal variables including maternal pre-pregnancy BMI were self-reported, and reporting bias cannot be ruled out. As national estimates revealed that women tend to underreport BMI by an average of 1.1 kg/m^2^, we believe the influence of reporting bias on the present analyses will be small [54]. It’s worth noting that despite BMI *z*-score being widely used to classify overweight and obesity in children, it does not account for body composition (lean and fat mass). Similarly, rapid weight gain does not differentiate increases in lean or fat mass. Future studies including the measurements of body composition will be desirable.

### Implications

Our study extends understanding of the early origins of childhood overweight and the relative contributions of prenatal and postnatal factors. The assessment of total, direct and indirect effects provide unique insights on potential pathways underlying each factor and childhood overweight outcomes. This study revealed complex relationships among maternal pre-pregnancy BMI, infant birth weight, breastfeeding duration, RWG in infancy and early childhood overweight outcomes. The study findings offer new evidence informing when to start preventive interventions and where to prioritize intervention efforts. The current study highlights early postnatal period or infancy as a critical window for overweight prevention in early childhood. RWG in infancy revealed the strongest associations with overweight outcomes in early childhood and was a significant mediator underlying the association between breastfeeding duration and childhood overweight outcomes. Overweight prevention interventions should focus on prevention of RWG in infancy and is likely to produce the most significant effects. Moreover, infant birth weight was also influential and was identified as a mediator underlying several pathways. Early overweight prevention interventions are also warranted before conception. Despite showing weaker associations, maternal pre-pregnancy BMI was the most consistent factor that showed direct associations with infant birth weight, breastfeeding duration, and childhood overweight outcomes. Maintaining healthy pre-pregnancy body weight should also be encouraged.

## Conclusion

Maternal pre-pregnancy BMI, infant birth weight, breastfeeding duration and RWG in infancy were interrelated and act in concert to impact overweight outcomes in early childhood via several direct and/or indirect pathways. RWG in infancy demonstrated the strongest direct positive associations with childhood overweight outcomes followed by infant birth weight and maternal pre-pregnancy BMI. The direct inverse associations between breastfeeding duration and childhood overweight outcomes were fully explained by RWG during infancy. Taken together, future childhood overweight prevention strategies should focus on prevention of RWG during infancy via promoting longer duration of breastfeeding. Given maternal pre-pregnancy BMI was implicated in several pathways lead to childhood overweight, encouraging women of childbearing age to maintain a healthy body weight during prenatal period is also crucial for prevention of childhood overweight.

## Supplementary information

Supplementary result table

Supplemetary file for raw result tables

## Data Availability

The datasets analyzed during the current study are not publicly available due ethical restrictions but are available from the corresponding authors along with relevant study chief investigators on reasonable request.
