# Supplementary material for: Understanding the pathways between prenatal and postnatal factors and overweight outcomes in early childhood: a pooled analysis of seven cohorts
Source: Int J Obes (Lond). 2023 Apr 3;47(7):574–82. doi: 10.1038/s41366-023-01301-9 (PMC10299910; doi:10.1038/s41366-023-01301-9)
Supplement: Supplementary file 1 — Supplementary result table [file 41366_2023_1301_MOESM1_ESM.docx]

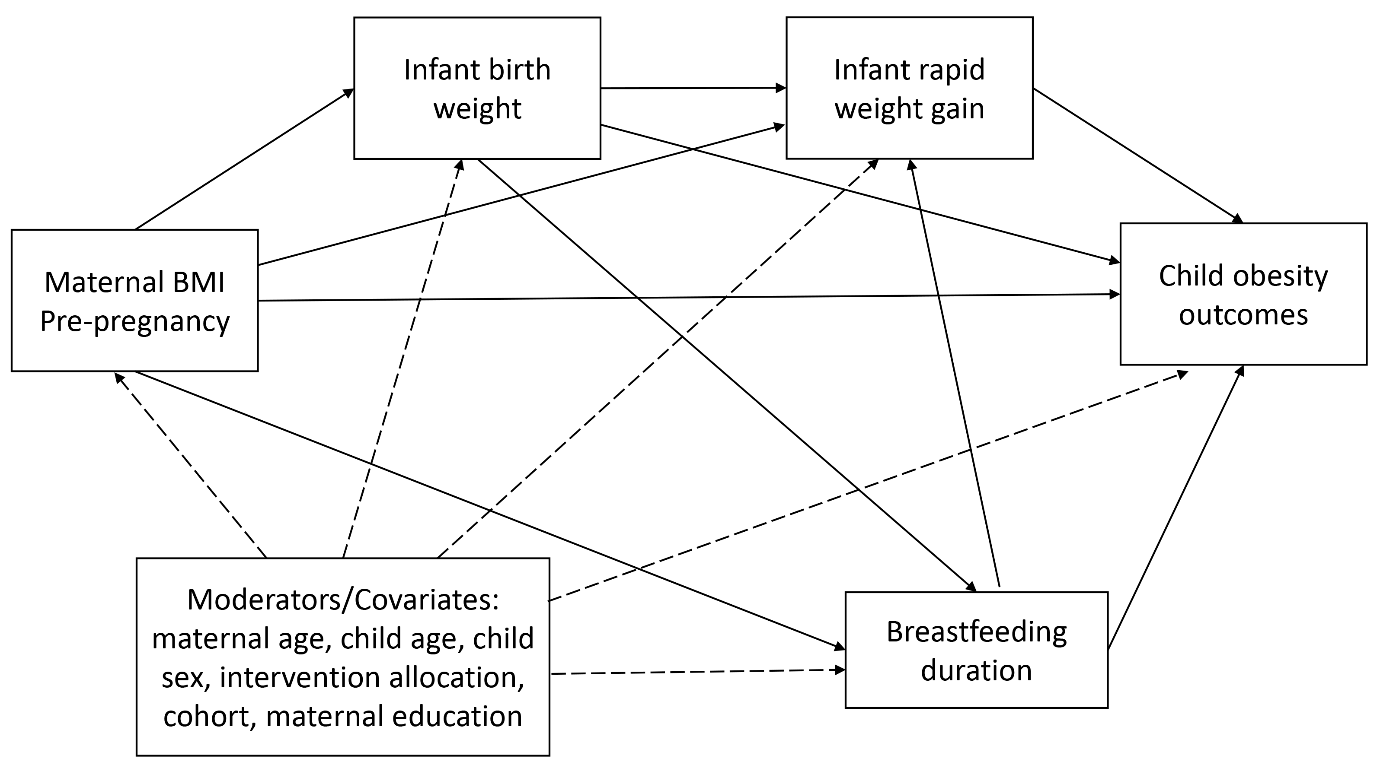


Supplementary Figure 1. Hypothesized pathway linking prenatal, postnatal and childhood obesity outcomes.

Supplementary Table 1. Total, direct and indirect effect among the pathways between prenatal (maternal pre-pregnancy BMI) and postnatal factors (infant birth weight, infant rapid weight gain, breastfeeding duration) and BMI z-score in early childhood: pooled effect from six Australian and New Zealand cohorts.

|  | β or Odds ratio | 95%CI | | P-value |
| --- | --- | --- | --- | --- |
| Maternal pre-pregnancy BMI → Child BMI z-core | | |  |  |
| Total effect | 0.06 | 0.04 | 0.08 | <0.001 |
| Direct effect | 0.04 | 0.03 | 0.05 | <0.001 |
| Indirect effect |  |  |  |  |
| via infant birth weight | 0.01 | 0.01 | 0.002 | <0.001 |
| via infant rapid weight gain | 0.02 | 0.002 | 0.03 | 0.03 |
| via breastfeeding duration | -0.005 | -0.01 | 0.001 | 0.10 |
|  |  |  |  |  |
| Maternal pre-pregnancy BMI → Rapid weight gain in infancy (yes vs no) | | | | |
| Total effect | 1.03 | 1.00 | 1.06 | 0.06 |
| Direct effect | 1.02 | 1.00 | 1.04 | 0.03 |
| Indirect effect |  |  |  |  |
| via infant birth weight | 0.96 | 0.94 | 0.97 | <0.001 |
| via breastfeeding duration | 1.05 | 1.03 | 1.08 | <0.001 |
|  |  |  |  |  |
| Maternal pre-pregnancy BMI → Breastfeeding duration (≥6 vs < 6 months) | | | | |
| Total effect | 0.94 | 0.92 | 0.95 | <0.001 |
| Direct effect | 0.93 | 0.91 | 0.95 | <0.001 |
| Indirect effect |  |  |  |  |
| via infant birth weight | 1.01 | 1.00 | 1.01 | 0.001 |
|  |  |  |  |  |
| Infant birth weight→ Child BMI z-score | |  |  |  |
| Total effect | -1.36 | -1.66 | -1.06 | <0.001 |
| Direct effect | 0.58 | 0.51 | 0.66 | <0.001 |
| Indirect effect |  |  |  |  |
| via infant rapid weight gain | -1.97 | -2.28 | -1.65 | <0.001 |
| via breastfeeding duration | 0.02 | -0.01 | 0.05 | 0.13 |
|  |  |  |  |  |
| Breastfeeding duration ≥6mo → Child BMI z-score | | |  |  |
| Total effect | -0.47 | -0.68 | -0.26 | <0.001 |
| Direct effect | 0.07 | -0.01 | 0.15 | 0.08 |
| Indirect effect |  |  |  |  |
| via infant rapid weight gain | -0.54 | -0.73 | -0.35 | <0.001 |

Adjusted for cohort, ages at infancy and early childhood assessments, maternal age, child sex, intervention allocation and maternal education. β-coefficients are presented for continuous outcome (BMI z-score), and odds ratios are presented for categorical outcomes. CI: confidence interval.

Supplementary Table 2. Total, direct and indirect effect among the pathways between prenatal (maternal pre-pregnancy BMI) and postnatal factors (infant birth weight, infant rapid weight gain, breastfeeding duration) and overweight status in early childhood: pooled effect from six Australian and New Zealand cohorts.

|  | Odds ratio | 95%CI | | P-value |
| --- | --- | --- | --- | --- |
| Maternal pre-pregnancy BMI → Child overweight status (yes vs no) | | | | |
| Total effect | 1.14 | 1.09 | 1.19 | <0.001 |
| Direct effect | 1.08 | 1.06 | 1.10 | <0.001 |
| Indirect effect |  |  |  |  |
| via infant birth weight | 1.02 | 1.01 | 1.03 | <0.001 |
| via infant rapid weight gain | 1.04 | 1.00 | 1.07 | 0.03 |
| via breastfeeding duration | 1.00 | 0.98 | 1.02 | 0.67 |
|  |  |  |  |  |
| Maternal pre-pregnancy BMI → Rapid weight gain in infancy (yes vs no) | | | | |
| Total effect | 1.03 | 1.00 | 1.06 | 0.06 |
| Direct effect | 1.02 | 1.00 | 1.04 | 0.03 |
| Indirect effect |  |  |  |  |
| via infant birth weight | 0.96 | 0.94 | 0.97 | <0.001 |
| via breastfeeding duration | 1.05 | 1.03 | 1.08 | <0.001 |
|  |  |  |  |  |
| Maternal pre-pregnancy BMI → Breastfeeding duration (≥6 vs < 6 months) | | | | |
| Total effect | 0.94 | 0.92 | 0.95 | <0.001 |
| Direct effect | 0.93 | 0.91 | 0.95 | <0.001 |
| Indirect effect |  |  |  |  |
| via infant birth weight | 1.01 | 1.00 | 1.01 | 0.001 |
|  |  |  |  |  |
| Infant birth weight→ Child overweight status (yes vs no) | | | | |
| Total effect | 0.06 | 0.02 | 0.13 | <0.001 |
| Direct effect | 3.33 | 2.53 | 4.37 | <0.001 |
| Indirect effect |  |  |  | <0.001 |
| via infant rapid weight gain | 0.02 | 0.01 | 0.04 | <0.001 |
| via breastfeeding duration | 1.02 | 0.93 | 1.11 | 0.68 |
|  |  |  |  |  |
| Breastfeeding duration (≥6 vs < 6 months)→ Child overweight status (yes vs no) | | | | |
| Total effect | 0.34 | 0.21 | 0.57 | <0.001 |
| Direct effect | 1.06 | 0.81 | 1.39 | 0.67 |
| Indirect effect |  |  |  |  |
| via infant rapid weight gain | 0.32 | 0.21 | 0.51 | <0.001 |

Adjusted for cohort, ages at infancy and early childhood assessments, maternal age, child sex, maternal education and intervention allocation. Odds ratios are presented for categorical outcomes; CI: confidence interval.
